# Investigation of Different Winemaking Protocols to Mitigate Smoke Taint Character in Wine

**DOI:** 10.3390/molecules27051732

**Published:** 2022-03-07

**Authors:** Anita Oberholster, Yan Wen, Sandra Dominguez Suarez, Jesse Erdmann, Raul Cauduro Girardello, Arran Rumbaugh, Bishnu Neupane, Charles Brenneman, Annegret Cantu, Hildegarde Heymann

**Affiliations:** Department of Viticulture & Enology, University of California Davis, One Shields Avenue, Davis, CA 95616, USA; yanwe@ucdavis.edu (Y.W.); sdsuarez@ucdavis.edu (S.D.S.); jnerdmann@ucdavis.edu (J.E.); rgirardello@ucdavis.edu (R.C.G.); acrumbaugh@ucdavis.edu (A.R.); bneupane@ucdavis.edu (B.N.); cabrenneman@ucdavis.edu (C.B.); acantu@ucdavis.edu (A.C.); hheymann@ucdavis.edu (H.H.)

**Keywords:** wine, volatile phenols, smoke, yeast, oak, fermentation temperature, sensory

## Abstract

There is an increase in the levels of volatile phenols in wine made with smoke-impacted grapes. These compounds are present in wood smoke resulting from the pyrolysis (thermal decomposition) of lignin and at high levels give overpowering smoky and ashy characters to a wine. This research aimed to compare all the suggested wine mitigation strategies that evolved from prior research using smoke-impacted grapes under identical winemaking conditions except for the parameter under investigation. Cabernet Sauvignon grapes were received from three areas with varying amounts of smoke exposure in Northern California. Gas chromatography combined with mass spectrometry (GC-MS) and descriptive analyses were performed to correlate the volatile phenol composition to smoke taint characteristics. The winemaking variables investigated were the use of different fermentation yeasts, oak additions, and fermentation temperatures. Among other attributes, smokiness and ashy aftertaste were significantly different among the wines, showing a clear difference between the wines made from smoke-impacted fruit and the control wines made from non-impacted fruit. Findings indicate that mitigation strategies during red wine fermentation have a limited impact on the extraction of smoke-taint markers and the expression of smoke-taint sensory characteristics.

## 1. Introduction

In recent years, the intensity and frequency of wildfires have increased in many countries, including Southern Europe, Australia, Canada, and the United States [1,2]. Wildfires release large amounts of gaseous pollutants (e.g., CO_2_, nitrogen oxides), polycyclic aromatic hydrocarbon, and volatile organic compounds into smoke, including volatile phenols [3,4,5]. The derived organic compounds can impact a larger area through the aerial transport of smoke than the initial combustions. Since 2003, smoke taint has been widely identified, causing quality problems in wines made from grapes grown near wildfire areas [6]. Some wines produced from smoke-affected grapes are dominated by unpleasant sensory attributes, such as smoky, burnt, and lingering ash [7,8,9,10,11], resulting in large economic losses for the grape and wine industries. Several research groups have highlighted that the expression and intensity of smoke-related flavors are mainly related to chemical differences of lignin-derived volatile phenols (VPs) [7,12]. Previous studies determined the concentration of guaiacol and 4-ethylguaiacol as chemical indicators of smoke-taint [13,14]. However, more recent studies have shown that other volatile phenols are also significantly associated with smoke flavors, especially ashy aftertaste. These VPs include cresols, phenol, syringol, and their substituted phenols, constituting a group of chemical markers for smoke-tainted wines [9,15].

The VPs released in the air spread through natural diffusion and are adsorbed through the berry cuticle and into the grape, where they are quickly glycosylated as mono, di, and triglycosides [16]. During winemaking, both the free and bound volatile phenols extract from the skins into the wine, further releasing free VPs via enzymatic and/or acidic hydrolysis during fermentation and bottle aging [14,17,18]. Research has also shown that enzymes in the saliva can break down the glycosidic bonds of volatile precursors, releasing smoke taint aroma in the mouth [19]. Currently, even when measuring the full panel of free and total or individual bound volatile phenols, it is not possible to accurately predict the extent of the smoke impact on a particular wine matrix [7]. 

Previous investigations indicated that different winemaking processing methods may be used to minimize the impact of smoke-derived compounds [12]. In general, reducing skin contact and using yeast strains to maximize the fruity characters in the wine helped to decrease the apparent taint [20]. Additionally, the use of oak chips and ellagic tannin enhanced the complexity of the wines and decreased the perception of smoke-related attributes. However, it was advised to avoid the use of any barrel/oak toast profiles that may contribute to smoke characters in the wine. These recommendations are based on single studies using multiple grape varieties with different levels of smoke exposure. The success of these strategies is likely highly dependent on the wine matrix. 

During the 2017 wildfires, we harvested Cabernet Sauvignon grapes from Napa and Sonoma Counties in California and made wines using different recommended winemaking protocols to mitigate the impact of volatile phenols, such as different yeast selections, oak and/or ellagic tannin additions, and fermentation temperatures to investigate the efficacy of these strategies on Cabernet Sauvignon smoke-impacted grapes.

## 2. Results

### 2.1. Chemical Analysis

The basic chemical composition of the wines at bottling is shown in Table 1. There were small but significant differences among treatments in all measured parameters (α = 0.05), but only the differences in alcohol content and residual sugar are deemed important to have a sensory impact [21]. In general, differences in ethanol content were driven by sugar content differences in the grapes coming from the three different vineyards (Supplementary data, Table S1). 

In Table 2, the total (free and bound) volatile phenol composition of the different wine treatments area shown. Free to total volatile phenol ratios were consistent and showed similar trends within wine treatments. In panel 1 wines, >85% of the VPs were in the free form, potentially due to late-season exposure to smoke. Additionally, the smoke impact in grapes from the Oakville AVA was relatively low, and the VP composition of the wines was only determined at the time of sensory three to six months after bottling. However, panel 2 wines showed a higher contribution from bound VPs, and although PCAs indicated a similar correlation between total and free VPs, the individual bound and free VP data are provided in Supplementary Tables (Tables S3 and S4). Most volatile phenols measured were significantly different among the various wine treatments. In general, the yeast treatments did not result in large changes among the treatments. D80 wine treatments contained the lowest amounts of volatile phenols, with D254 containing the highest. Overall, the impact of yeast was low when only evaluating the volatile phenol composition, and no significant impact due to yeast treatment was observed in the amount of free VPs released from bound precursors. Similarly, the addition of oak alternatives did not have a large impact on the volatile phenol composition of the wines. Nobile Base (NB) contributed slightly more volatile phenols to the ferment than Nobile Fresh (NF), although syringol concentrations were in fact lower than the control treatment (EC1118). The Quertanin addition (ellagic tannin) did not contribute significant amounts of volatile phenols to the wines. 

Two vineyard blocks at the Oakville Experimental Station in Oakville, AVA were harvested (OFV6, block 6, and EC1118 control treatment from block 9). These grapes were exposed to similar environmental conditions, but contained significantly different amounts of volatile phenols, indicating the potential impact of vine age and plant vigor (clone, rootstock, and trellis systems were similar). Block 6 was planted in 1993, whereas block 9 was planted in 2014. Although the wines made from non-smoke-exposed grapes contained the lowest amounts of volatile phenols, the comparative increase in the smoke-exposed grapes was relatively modest, potentially indicating a low impact due to smoke exposure. Although wildfires move within a mile of the vineyard, the wind direction was mostly away from the vineyard. 

The different fermentation temperatures at the seven-day skin contact did not have a significant impact on the final volatile phenol content. Only for the 10 °C treatment, where grapes were cold soaked for seven days, pressed, and then fermented, were there volatile phenols (guaiacol and syringol) significantly lower compared to the control treatment (AV_25 °C). The extraction of VPs at different temperatures was followed as shown in Table 3. Free and bound VPs were extracted at a constant rate irrespective of temperature (data not shown). Furthermore, our data indicated that after three days of skin contact, independent of temperature, between 78 to 91% of the VPs were extracted compared to the final wines and that pressing did not have a significant impact on the amount of VPs in the wine after at least 7 days of skin contact. Furthermore, the treatment applied to the Stag’s Leap District grapes were also unsuccessful, with the wines treated with pectolytic enzymes and ellagic tannin (ST_E+T) having, in general, similar or higher levels of all the measured volatile phenols compared to the control wine made using standard experimental winemaking protocols (ST_C). The reference wines for panel 2 were from Oakville, AVA, harvested before and after smoke exposure, with an increase in volatile phenol concentrations for all measured compounds in the wines made from smoke-exposed versus not-smoke-exposed grapes. 

### 2.2. Sensory Analysis

The trained judges from panel 1 evaluated 30 attributes corresponding to aroma, taste, and mouthfeel for each of the wines made from grapes from Oakville, AVA in triplicate (unsmoked, EC1118, BDX, D80, D254, NB, NF, and QT) (Supplementary Table S5). Multivariate analysis of variance (MANOVA) indicated that there were no significant differences among the wine treatments (α = 0.05) for the 30 sensory attributes evaluated and, therefore, the subsequent ANOVAs need to be evaluated with caution. ANOVA showed that out of those thirty attributes, only nine were considered significantly different among the wine treatments when analyzed individually. The attribute means and Fisher’s least significant difference (LSD) for the significant attributes are shown in Table 4. The ‘smoke’ aroma attribute was surprisingly not significantly different among the wine treatments, even though a non-smoke-exposed control treatment was included in the evaluation. All wines were rated smoky at a low level (mean of 1.3) indicating that a smoky aroma was not a major attribute for any of the wines evaluated, but also that there was a small amount of carryover among the samples, even though a 1 min wait time was enforced between samples. In subsequent studies, we determined that longer wait times are needed when evaluating smoke-impacted wines. This was confirmed by a recent study by Fryer et al. [22] that found a 2 min waiting time to be optimal. Our own investigation indicated that a 90 s wait time using a 0.5% pectin rinse was sufficient for low to medium smoke-impacted wines. Nevertheless, an ashy aftertaste was found to be one of the nine significantly different attributes.

**Figure 1:**
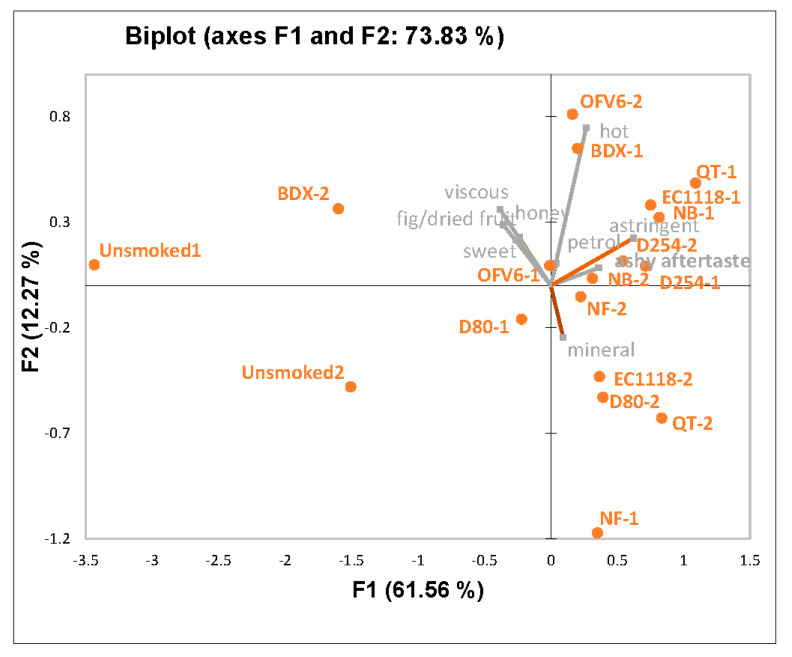
PCA biplot of all wine treatments from panel 1.

A principal component analysis (PCA) was performed to better visualize differences among the wines (Figure 1). The first two components (PCs) were able to explain approximately 74% of the data. ‘Fig/dried fruit’ and ‘honey’ aromas, ‘sweet’ tastes, and ‘viscous’ mouthfeel correlated positively with each other and correlated negatively to attributes such as ‘ashy aftertaste’ aroma and ‘astringent’ mouthfeel (Supplementary Table S6). The non-smoke-exposed control wines (Unsmoked) were rated lower for ‘ashy aftertaste’, while they were also considered to be much higher in ‘sweet’ taste and ‘fig/dried fruit’ aroma. The low ‘ashy aftertaste’ rating does indicate some carryover; however, the panelists were still able to distinguish among the wines based on this attribute.

**Figure 2:**
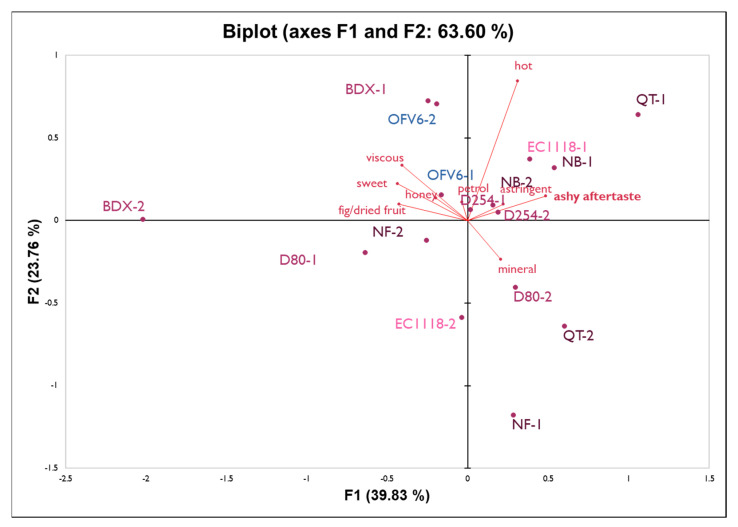
PCA biplot for yeast, oak, and tannin treatment in wines from Oakville, AVA.

Wines made from fruit not exposed to smoke were seen as very different from the rest of the wine treatments, despite being made from grapes from the same vineyard block. Thus, another PCA was created without the unsmoked controls to better evaluate the differences perceived by the judges for the treatments applied to the wines from smoke-exposed fruit. The first two components of the PCA shown in Figure 2 explain 63% of the variance.

The third dimension was constructed, and although it resulted in a better representation of the attributes ‘petrol’ and ‘astringent’, the first two dimensions were chosen as it explained more of the variance and the rest of the attributes were better represented. This third dimension (not shown) explains another 13% of the variance in the data and was able to show that wines fermented with the yeast D254 were more closely correlated with the ‘astringent’ and ‘petrol’ aroma characteristics. As mentioned previously, the wines fermented with EC1118 are considered the reference treatment (standard winemaking practices) for the purpose of this study. Variability between fermentation replicates does not allow clear treatment interpretation. Wines from the Oakville Experimental Station block 6 (OFV6), which were made under the standard experimental winemaking conditions with the yeast EC1118, can be compared with OFV9 as this block came from the same vineyard and received a similar amount of smoke exposure. OFV6 wines were perceived to be sweeter and fruitier, with a decreased ‘ashy aftertaste’. The addition of oak chips (NB, NF) and an ellagic tannin (QT) during fermentation did not have a clear impact on the sensory perception of the wine compared to the control (EC1118). 

The trained judges from panel 2 evaluated a total of 27 attributes corresponding to aroma, taste, and mouthfeel for each of the wines made from grapes from the Stag’s Leap District and Alexander Valley AVAs in triplicate. MANOVA revealed significant differences among wine treatments (α = 0.05) for the sensory attributes evaluated (Supplementary Table S7). ANOVAs showed that 15 attributes were significantly different among all wine treatments. For some of these attributes, there was a significant wine-by-judge and wine-by-replicate interaction and, therefore, a pseudo-mixed model was applied (Supplementary Table S8). Adjusted F values indicated that for ‘dark fruit’ and ‘leather’ aromas and ‘astringency’ mouthfeel, the effect of the wine treatment is more important than the individual interactions. ‘Dark fruit’, ‘vanilla’, and ‘raisin/prune’ aromas were highly correlated, while mouthfeel attributes ‘hot’ and ‘astringent’ were negatively correlated to the ‘smoky’ aroma character (Supplementary Table S9). The attribute means and Fisher’s least significant difference (LSD) values for the significant attributes are shown in Table 5.

**Figure 3:**
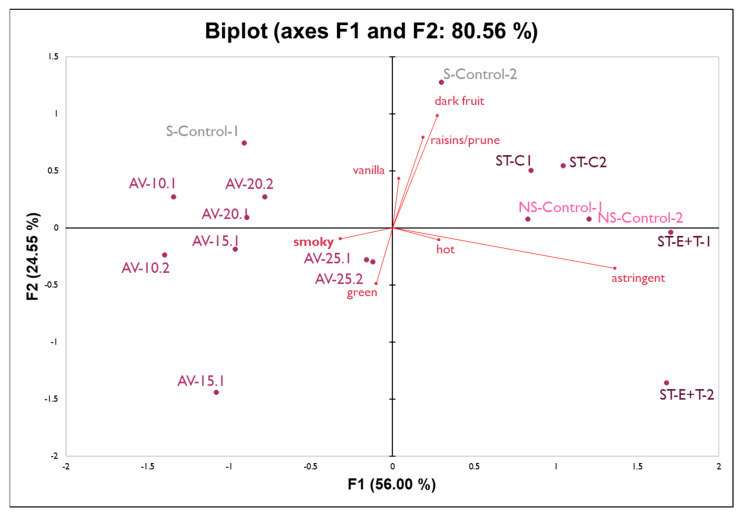
PCA biplot of all wine treatments from panel 2.

Although the non-smoke-exposed wines were rated as ‘smoky’ by panelists, they were rated lowest for the ‘smoky’ aroma attribute while also considered to be on the higher end of attributes such as ‘dark fruit’, ‘vanilla’, and ‘raisin/prune’. To visualize the differences among the wines for the eight significantly different attributes, PCA with a covariance matrix was performed, where the first two principal components (PC) were able to explain 80% of the data (Figure 3). The first component of the PCA is mainly driven by astringency on the horizontal axis while the second component is driven positively by the fruity aromas and negatively by green and leather characteristics. 

There is a clear clustering of the wines made from grapes from different vineyards, as Figure 3 shows the wines from fruit from Alexander Valley (AV) on the left side and wines made from fruit from Stag’s Leap District (ST) on the right side of the PCA. The non-smoked-exposed (NS_Control) and the smoke-exposed (S_Control) control wines were made from fruit from Oakville, AVA. It appears that wines made from the Stag’s Leap District, AVA were less ‘smoky’, suggesting that the smoke impact in Alexander Valley was greater. The Pocket Fire in Sonoma County was closer to the vineyard in Alexander Valley [23] than the fires were to the Napa County vineyards. Unfortunately, ‘ashy aftertaste’ was not significantly different among the wine treatments due to the significant wine-by-judge interaction. This could be a result of carryover due to only 90 s in wait time with a water rinse between wines. Different fermentation temperatures (15, 20, and 25 °C, and a cold soak at 10 °C) did not have a significant impact on smoke expression in the resulting wines, while the enzyme and tannin addition treatment seemed to enhance the perception of astringency of the wines. 

**Figure 4:**
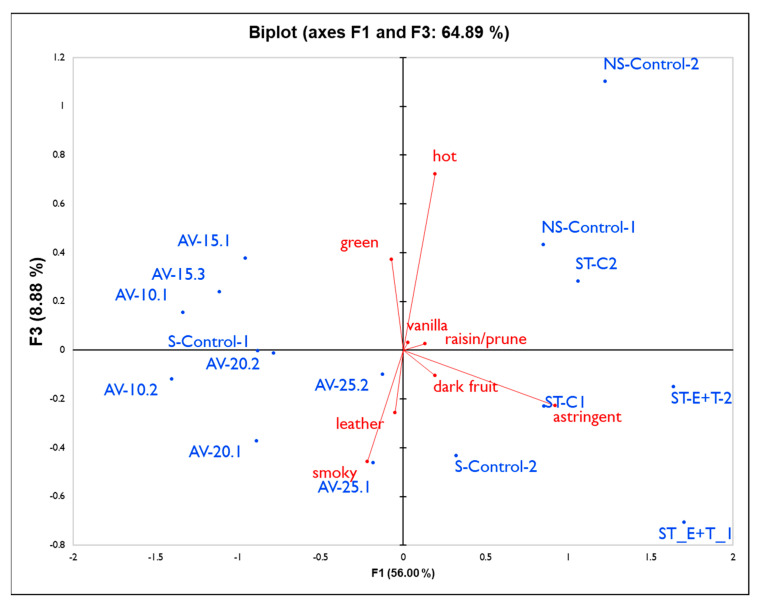
PCA biplot with the first and third PC for all wine treatments from panel 2.

Figure 4 shows the first and third components, explaining another 9% of the data. Wines from the Stag’s Leap District grapes, especially the treatment with an enzyme and tannin addition (ST_E+T), were rated as being more astringent and not very smoky, while the non-smoke-exposed control wines (NS-Control) were rated as ‘hot’ and the lowest values for ‘smoky’ character. The wines that were cold soaked at 10 °C then pressed and fermented following a white winemaking protocol (AV_10) proved to be better balanced, with some fruit, a mild green character, and lower astringency, although they were still perceived as smoky. Wines fermented at 15 °C correlated with green character, while higher fermentation temperatures did not, and, in general, wines from the Alexander Valley were rated as less astringent than the rest of the wines evaluated. Overall, the wines used as controls for both smoke- and non-smoke-exposed fruit from the Oakville Experimental Station (S-Control and NS-Control) revealed a more intense vanilla character, while the control wines from Stag’s Leap District (ST_C) were perceived to have a higher dark fruit aroma when plotting the first against the fourth component of the PCA (results not shown).

## 3. Discussion

In general, the use of different yeasts for the fermentation of smoke-impacted grapes resulted in only small differences in the wine’s final VP composition. Although it is known that yeasts vary in their ability to cleave glycosidically bonded compounds, no consistent impact due to yeast strain was observed on free versus bound VP ratios. This may be due to the relatively small percentage of VPs that were in the bound form in these grapes. The descriptive analysis did indicate a lower perception of ‘ashy aftertaste’ for D80 wines, which contained the lowest amount of total VPs. However, there was a large difference between the D80 fermentation replicates for this attribute. BDX wines contained similar VP concentrations compared to the controls but were rated as fruitier, sweeter, and less ashy. This is potentially due to a masking effect of the fruity aromas [24,25]. Additionally, BDX had the highest concentration of residual sugar (6.88 g/L), and research has shown that residual sugar above 3 g/L can inhibit the bacterial enzymes in a taster’s saliva that can release glycosylated VPs and contribute to the ashy aftertaste [26]. Ristic et al. [20] evaluated the use of different yeasts, including BDX, on smoke-impacted Grenache grapes. However, only small-scale fermentations (4.5 kg each) were performed on frozen grapes at 20 °C for 6 days of skin-contact time. Under these conditions, BDX was one of the treatments that were rated the most smoky and ashy. The two smoke control treatments (OFV6 and EC1118) and D254 contained significantly higher alcohol levels, which can suppress the fresh fruit character and astringent mouthfeel as well as contribute to the perception of ‘sweet’, ‘viscous’, and ‘hot’ [27]. The impact was, however, not clear, with a positive correlation with the perception of ‘hot’ and a potential decrease in fruity character for treatments EC1118 and D254. 

Given that oak products such as oak chips and hydrolyzable tannins can be sources of free VPs in wines [28], it was expected that wine treatments fermented with oak additions will have higher values of these compounds. However, VP data did not really reflect this, with mostly small increases in creosol (4-methylguaiacol) and 4-methylsyringol observed. The lower-than-expected total VP concentrations could be due to a small percentage of glycoconjugates being absorbed by the oak and tannin additions as hypothesized by Ristic et al. [20]. The authors also found that the added complexity from the oak and the uplifting of fresh fruity and sweet aromas helped mask the smoke character in Shiraz wines [20]. In our study, the addition of oak chips (NB, NF) and an ellagic tannin (QT) during fermentation did not have a clear impact on the sensory perception of the wine compared to the control (EC1118). 

Both phenolic compounds and volatile phenols are located primarily in the grape skins, and they are extracted during fermentation from the skins during red wine making. Fermentation temperature is a key factor for phenolic extraction, and it was hypothesized that it would also play an important role in the extraction of volatile phenols and their glycoconjugates. Higher temperatures increase the permeability of the hypodermal cells, allowing for an increase in diffusion and solubility of these compounds in the wine [29]. Hence, it was investigated whether lower temperatures can significantly reduce the extraction of volatile phenols and minimize the smoke taint expression in the wines. Ristic et al. [20] determined that there was a significantly less smoky character in rosé wine (three days cold soak at 0 °C and fermentation at 15 °C) compared to the equivalent red wine from the same fruit. However, previous studies have demonstrated that the extraction of almost half of the volatile phenols available occurs within the first three days of fermentation [6]. Our own data show an even higher extraction rate ranging between 78 and 9% of the final VP concentration in the wines after pressing. Results also indicated that skin contact time was more important than temperature. The VP composition of the different fermentation temperature treatments indicates no significant differences among the 15, 20, and 25 °C fermentation with seven days of skin contact. Only the cold-soak treatment at 10 °C contained about 20% less VPs compared to the control (25 °C). However, panelists perceived these wines as being similar in ‘smoky’ character. This may be partly because although the cold soak treatment contained less VPs, the rosé wine also has a simpler matrix that could allow the smoky character to be more visible. The treatment investigating the potential of using pectolytic enzymes with tannin additions to extract and stabilize color early during fermentation to limit skin contact indicated that pectolytic enzymes not only promote the extraction of anthocyanin and other skin phenolics [30], but also the VPs present in the skin. Thus, this treatment is not seen as a viable option to decrease VP extraction. 

Although some of the wine treatments showed changes in the concentration of certain VPs, it is important to consider that these compounds have a synergistic and matrix effect, and hence, correlations cannot always directly be made with sensory characteristics. To that end, a multiple factor analysis (MFA) was performed. The correlation map of the variables for panel 1 showed that all measured VPs correlated with an ‘ashy aftertaste’, whereas all measured VPs except for 4-ethylguaiacol correlated with a ‘smoky’ aroma in panel 2 (Supplementary Figures S1 and S2). Thus, for the wine treatments investigated, the measured VP correlated with the main sensory attributes that indicate the smoke impact.

## 4. Materials and Methods

### 4.1. Chemicals and Reagents

The following chemicals were of analytical reagent grade. Eugenol, 4-methylsyringol, 4-ethylguaiacol, 4-ethylphenol and hydrochloric acid (HCl) were purchased from Sigma-Aldrich (St. Louis, MO, USA). Guaiacol, *o*-cresol, *p*-cresol, and *m*-cresol were obtained from TCI America (Portland, OR, USA). 4-methylguaiacol and syringol were purchased from Alfa Aesar (Tewksbury, MA, USA). Deuterated standards d3-guaiacol, d3-4-methylguaiacol, d7-*o*-cresol, d7-*p*-cresol, d7-m-cresol, d5-4-ethylguaiacol, and d4-4-ethylphenol were obtained from CDN Isotopes (Pointe-Claire, QC, Canada) and d6-syringol was purchased from EPTES (Vevey, Switzerland). Solvents ethyl acetate (EtOAc) and pentane were of HPLC grade and purchased from Millipore Corporation (Darmstadt, Germany) and Sigma-Aldrich (St. Louis, MO, USA), respectively. HPLC-grade ethanol used in this study was from Sigma-Aldrich (St. Louis, MO, USA). Water was produced by a Milli-Q Element system (Millipore, Rockville, MD, USA). 

### 4.2. Grapes and General Winemaking 

The grapes used for this study were sourced from *Vitis vinifera* L. *cv*. Cabernet Sauvignon from three different vineyards with different amounts of smoke exposure located in the Oakville American Vineyard Area (AVA), Alexander Valley AVA, and Stags Leap District AVA in California (Supplementary Table S1). Fermentations were carried out in triplicate using 30-gallon stainless-steel research fermenters at the UC Davis Research and Teaching Winery as described in Lerno et al. [29]. Briefly, the standard winemaking procedure was as follows, unless specifically stated otherwise. Fifty mg/L sulfur dioxide was added in the form of a 15% potassium metabisulfite solution directly after destemming and crushing. The day after fruit processing, 25 g/hL Fermaid K (Lallemand, Santa Rosa, CA, USA) was added, and the diammonium phosphate (DAP) was needed to achieve total yeast assimilable nitrogen levels of 300 mg/L. DAP was added in two installments, the first prior to inoculation and the second after one-third of sugar depletion. The musts were inoculated after a 24 h cold soak at 10 °C, with *Saccharomyces cerevisiae* strain Lalvin EC 1118 (Lallemand, Santa Rosa, CA, USA) unless stated otherwise, according to the manufacturer’s rehydration procedure. Fermentation temperatures were controlled at 25 ± 1 °C unless specified differently, and all wines were pressed after 7 days of fermentation when dry (residual sugar <2 g/L). Cap management was performed by pumping one tank volume of wine from the bottom of the tank over the fermentations through an irrigator twice a day. Wines were pressed using a Cypress Semiconductor hydraulic press. The resulting wines were inoculated for malolactic fermentation (MLF) with *Viniflora Oenococcus oeni* (Chr. Hansen A/S, Hørsholm, Denmark) according to the manufacturer’s protocol. MLF was monitored weekly by following the decrease in malic acid concentration by enzymatic analysis (Gallery Automated Photometric Analyzer, Thermo Fisher Scientific, Waltham, MA, USA). After MLF was completed, free sulfur dioxide was adjusted to 35 mg/L and the wines were sterile filtered (0.45 μm) and bottled in Bordeaux bottles with screw caps (Saranex/Transcendia, Franklin Park, IL, USA). All the bottled wines were stored at 14 °C until further analysis. 

### 4.3. Wine Treatments

The experimental design of the different winemaking treatments and respective treatment names are listed in Table 6. All fermentation treatments were performed in triplicate. 

#### 4.3.1. Yeast Strains

Smoke-exposed CS grapes in this trial were harvested from the Oakville Experimental Vineyard in Oakville (vineyard block 9), CA. Grapes were harvested on the 17 October 2017 after 10 days of smoke exposure from the Tubbs (Central LNU Complex) fire (Supplementary Table S2). Fermentations were performed with four different Saccharomyces cerevisiae yeast strains: EC 1118, Lalvin ICV D254, Lalvin ICV D80, and Enoferm BDX (Lallemand, Santa Rosa, CA, USA). The control grapes that were not exposed to smoke from the same vineyard were harvested ten days earlier just before the start of the fire (17 October 2017) and inoculated with the yeast strain EC 1118 as the control wine treatment (Unsmoked). From the same vineyard, an additional vineyard block (block 6) was harvested, but due to the limited grapes obtained, OFV6 was fermented as a smoke-exposed control with no winemaking treatments applied.

#### 4.3.2. Oak and Tannin Additions 

As with the yeast strain trial, CS grapes in this trial were harvested from the Oakville Experimental Vineyard in Oakville (block 9), CA, after 10 days of smoke exposure (harvest date 17 October 2017) (Supplementary Table S2). The first treatment investigated the addition of ellagic oak tannins (Quertanin, Laffort, St. Helena, CA, USA) at 0.5 g/L to the must at the start of fermentation. The second and third treatments entailed the addition of two types of French oak chips: Untoasted Nobile Base (NB) and lightly toasted Nobile Fresh (NF) (Laffort, St. Helena, CA, USA) at 5 g/L, respectively. These addition levels were according to the recommended dosage by the manufacturers and previous research conducted by Ristic and coauthors [20]. 

#### 4.3.3. Skin Contact Time and Maceration Enzyme

Smoke-exposed CS grapes in this treatment were harvested from the Stag’s Leap District in Napa Valley, CA, after two weeks of smoke exposure from the Atlas (Southern LNU Complex) fire (harvest date 23 October 2017) (Supplementary Table S2). In this trial, grapes were either fermented using the standard winemaking protocol as described previously (EC1118 control) or a commercial pectolytic enzyme HE Grand Cru (Laffort, St. Helena, CA, USA) was added at 35 g/ton during processing, followed by Tannin VR Supra (Laffort, St. Helena, CA, USA) addition at 300 mg/L to prevent oxidation. When residual sugar decreased to 5 Brix, 300 mg/L of Tannin VR Color (Laffort, St. Helena, CA, USA) was added to the same wine for color stabilization. The musts were pressed after 4 days of skin contact. This treatment is referred to as E+T due to the usage of enzymes and tannins (Table 6). 

#### 4.3.4. Fermentation Temperature

Grapes harvested from a CS vineyard in the Alexander Valley AVA in CA (AV) were deemed as more impacted due to 10 days of smoke exposure from the Pocket fire in 2017 and its proximity to the fires (harvest date 18 October 2017) (Supplementary Table S2). Four different treatments were applied to the must. The first treatment entailed a cold soak at 10 °C for seven days followed by pressing and fermentation at 25 °C of the resulting juice for seven days until dry (<2 g/L RS). The other three treatments were fermented at 15, 20, and 25 °C, respectively, following the general winemaking procedures described previously. Samples were taken daily to determine VP extraction rates at different fermentation temperatures.

### 4.4. Wine Analysis

The final wine chemical parameters were determined the day before bottling (Table 1). Ethanol content % (*v*/*v*) was measured with an analyzer (Anton Parr, Ashland, VA, USA), while the pH and titratable acidity (TA) were measured using an Orion 5-star pH meter (Thermo Fisher Scientific, Waltham, MA, USA) and a Mettler-Toledo DL50 titrator (Mettler-Toledo Inc., Columbus, OH, USA), respectively. The acetate, malate, and residual sugar (RS) were determined by enzymatic analysis using the Gallery automated analyzer (Thermo Fisher Scientific, Waltham, MA, USA).

### 4.5. Gas Chromatography-Mass Spectrometry (GC-MS)

Free and total volatile phenols (VP) in wine were analyzed by liquid–liquid extraction (LLE). The LLE protocol was modified from Noestheden et al. [31]. Five milliliters of grape homogenate or wine were spiked with deuterated internal standards at 20 µg/L, except d6-syringol, which was spiked at 40 µg/L. The mixture was extracted with 2 mL of EtOAc:pentane (1:1) for one hour after vortexing. After centrifugation, the organic layer was transferred to 2 mL glass vials for GC-MS analysis. Bottle duplicates were analyzed for all fermentation replicates, and all samples were analyzed in triplicate.

The amount of total volatile phenols (free and glycosidically bound VPs) was measured using the harsh-acid hydrolysis method with minor modifications [31,32]. Ten milliliters of wine samples spiked with deuterated internal standards (20 µg/L) were transferred to PTFE tubes with PTFE caps. Samples were acidified to pH 1 with concentrated HCl and heated to 100 °C for one hour. After hydrolysis, samples were LLE as described previously. 

Analyses were performed with an Agilent 7890B gas chromatograph equipped with a 5977B high-efficiency source (HES) mass spectrometer (Agilent Technologies, Santa Clara, CA, USA). A J&W DB-WAXetr capillary column was used (30 m × 0.25 mm i.d. × 0.25 μm thickness, Agilent, Santa Clara, CA, USA). Two microliters of the obtained LLE extraction were injected by a CTC Pal autosampler for all the analyses in splitless mode. The injection port temperature was set at 200 °C. The oven temperature started at 40 °C and held for five minutes, then raised to 220 °C at 6 °C/min, then finally increased to 250 °C at 50 °C/min and held at this temperature for seven minutes. The carrier gas helium was at a constant flow of 1 mL/min. The temperature of the ion source and transfer line were maintained at 230 °C and 250 °C, respectively. The mass spectra were collected in both scan and selective ion monitoring (SIM) modes with electron ionization. Both free and total VPs were quantified using the stable isotopic dilution analysis (SIDA) as described in Pollnitz et al. [28]. Stock solutions for VPs and deuterated internal standards were prepared in ethanol. Calibration solutions were freshly prepared before analysis by adding known amounts of VPs into a model wine (16% vol ethanol, 5 g/L potassium bitartrate, pH 3.75). 

### 4.6. Descriptive Analysis

The sensory profiles of the Cabernet Sauvignon wines were determined three to six months after bottling using descriptive analysis (DA) in the J. Lohr Wine Sensory Room, University of California, Davis, CA, USA [33]. The panelists were recruited via advertisement within the university and were selected based on their availability and interest. The study was approved by the Institutional Review Board of the University of California, Davis (IRB ID 1288072-1) and all panelists gave informed written consent. The panelists were not aware of the purpose of the study or how many different samples they were evaluating. Bench tasting of triplicate wine treatments revealed fermentation differences unrelated to the variable under investigation for some wine treatments. Thus, duplicate fermentations were selected for each treatment for sensory evaluation based on smell, taste, and mouthfeel. The selected wine treatment replicates were divided into two panels: Panel 1 evaluated all wine treatments from Oakville, AVA while panel 2 evaluated the wine treatments made from Stag’s Leap District and Alexander Valley, AVA fruit. Panel training consisted of nine one-hour training sessions over two weeks, where the judges generated appropriate attributes and reference standards for aroma, taste, and mouthfeel, and gained familiarity in recognizing and scoring the intensity of specific attributes. All wines were evaluated in triplicate by all the judges. Thirty milliliters of wine were poured into black tasting glasses (ISO-3591:1977) with an assigned randomized three-digit code to avoid biases caused by any possible differences in color. Sensory evaluation sessions took place in isolated sensory booths equipped with positive air flow, where the research wines were presented in a randomized block design. Wines were separated by a forced one-minute wait between samples where fresh water and unsalted Premium saltine crackers (Nabisco) were provided to cleanse the palate. The evaluation of the wines involved the rating of each individual attribute on a 10 cm unstructured line scale anchored by the words “low” and “high”. Sensory data were collected using FIZZ software (ver. 2.50, Biosystèmes, Couternon, France).

Panel 1 consisted of eleven judges who evaluated the yeast treatment wines made from Oakville, AVA fruit (Table 6) using 30 attributes in total (24 aroma, 3 taste, and 3 mouthfeel) (Supplementary Table S5). Six wines were evaluated per session over nine sessions in three weeks. Panel 2 consisted of twelve judges and analyzed the wines made from Stag’s Leap District and Alexander Valley, AVA fruit (Table 6) using 27 attributes (21 aroma, 3 taste, and 3 mouthfeel) (Supplementary Table S7). Since wildfires are unpredictable, there were no non-smoke-exposed controls from each one of these vineyards. Thus, CS grapes from the Oakville AVA, which were harvested before and after the wildfires, were used as general non-smoke-exposed (NS-Control) and smoke-exposed controls (S-Control) for comparison with the rest of the treatments. Due to poor air quality during the 2018 wildfires, the UC Davis campus was closed for two weeks during the training and formal evaluations of panel 2. This resulted in a loss of time and, therefore, eight wines per session were assessed during six sessions over a week and a half. However, given that some carryover was observed in the first panel, the evaluation sessions for this panel had a 90 s forced break between samples with a 10-min break after the first four wines. 

### 4.7. Statistical Analysis

Quantitative analysis of GC−MS data was conducted using the Mass Hunter Workstation software suite (version B.09.00, Agilent Technologies, Santa Clara, CA, USA). All the statistical analyses were performed using XLSTAT (2019, Addinsoft, New York, NY, USA). All chemical and sensory data were analyzed for statistical significance using multivariate analysis of variance (MANOVA) for the overall main treatment effect. If significant, univariate analyses of variance (ANOVA) measuring the effects of treatment and replicate using a pseudo-mixed-model test were used for all chemical data. ANOVA employing the effects of judge, treatment, and replicate with a pseudo-mixed model was used for the DA. Fisher’s least significant differences (LSD) were calculated among univariate mean values to assess significant differences. For the DA data, treatments were compared graphically using principal component analysis (PCA) on the mean data of the significant attributes only. The chemical and descriptive sensory data were related to one another using multiple factor analysis (MFA). Significant differences were assessed on a 5% significance level (*p* < 0.05). 

## 5. Conclusions

Different mitigation strategies in the winery were investigated in smoke-impacted Cabernet Sauvignon grapes from the 2017 wildfires in Northern California. The volatile phenol composition of the different wine treatments indicated that the use of different yeasts and oak additives have a small but variable impact and may vary depending on the grape varietal and level of smoke impact. Fermentation temperature had little impact, potentially due to the ease of extraction of VPs from grape skins. Therefore, the skin contact time will be a more important variable than fermentation temperature. The sensory panels were able to distinguish wines made from smoke-impacted fruit from those that were made from fruit with no smoke exposure. However, waiting times between wine evaluations need to be increased to avoid carryover effects. Findings indicate that mitigation strategies during red wine fermentation have a limited impact on the extraction of smoke-taint markers as well as the expression of smoke taint sensory characteristics, and therefore, further studies should focus on the amelioration of finished wines impacted by smoke. That said, synergistic impacts of some of the investigated winemaking protocols may make a difference for wines made from grapes with low smoke-exposure impact. Additionally, there are wine treatments with known efficacy in removing some of the free volatile phenols and reducing the impact of smoke exposure, such as activated carbon fining and reverse osmosis. New studies should investigate these and other treatment options further to improve their specificity. 

## Figures and Tables

**Table 1:** Basic chemical composition of all wine treatments at bottling (n = 6; α = 0.05).

Wine	Alcohol (%)	pH	TA (g/L)	Residual Sugar (g/L)
Panel 1 (Impact of yeast/oak)				
OFV6	16.68 ± 0.18 a	3.78 ± 0.02 e	6.18 ± 0.25 a	1.21 ± 0.45 b
EC1118	16.35 ± 0.11 a	3.75 ± 0.01 b	5.60 ± 0.24 c	1.01 ± 0.24 b
BDX	15.44 ± 0.40 c	3.78 ± 0.07 d	5.63 ± 0.11 c	6.88 ± 0.60 a
D80	15.89 ± 0.14 b	3.72 ± 0.01 bc	5.99 ± 0.02 ab	0.76 ± 0.29 bc
D254	16.63 ± 0.05 a	3.73 ± 0.02 bc	5.90 ± 0.03 abc	0.96 ± 0.23 b
NB	15.90 ± 0.01 b	3.80 ± 0.04 cd	5.62 ± 0.11 c	0.75 ± 0.04 bc
NF	15.74 ± 0.08 bc	3.77 ± 0.00 b	5.73 ± 0.18 bc	0.74 ± 0.08 bc
QT	15.89 ± 0.11 b	3.82 ± 0.00 a	5.63 ± 0.11 c	0.75 ± 0.03 bc
Unsmoked	14.72 ± 0.06 d	3.88 ± 0.03 a	5.94 ± 0.10 abc	0.42 ± 0.10 c
Panel 2 (Impact of fermentation temperature/maceration enzymes and tannin)				
AV_25	14.83 ± 0.47 cd	3.81 ± 0.01 d	5.40 ± 0.09 cd	0.44 ± 0.06 b
AV_20	14.92 ± 0.06 cd	3.82 ± 0.01 d	5.22 ± 0.05 d	0.49 ± 0.29 b
AV_15	15.14 ± 0.02 bcd	3.83 ± 0.01 d	5.47 ± 0.14 c	0.52 ± 0.06 b
AV_10	15.18 ± 0.29 bcd	3.64 ± 0.05 e	5.48 ± 0.08 c	0.41 ± 0.35 b
ST_C	15.49 ± 0.14 b	3.96 ± 0.01 a	4.91 ± 0.02 e	0.38 ± 0.01 b
ST_E+T	15.30 ± 0.17 bc	3.85 ± 0.01 b	5.42 ± 0.08 cd	0.53 ± 0.01 b
S_Control	15.51 ± 0.16 d	3.71 ± 0.02 d	6.42 ± 0.16 a	0.61 ± 0.18 b
NS_Control	14.30 ± 0.02 a	3.70 ± 0.03 c	6.21 ± 0.21 b	0.24 ± 0.02 a

Notes: Volatile acidity measured as acetic acid was below 0.5 g/L for all samples. Significance indicated when letters following values are different within a panel for chemical measurement. Wine treatment replicates were not significantly different.

**Table 2:** Total (free + bound) volatile phenol profiles of different wine treatments (n = 6, α ≤ 0.05). All con-centrations are in μg/L.

Wine	Guaiacol	Creosol	*o*-Cresol	4-Ethylguaiacol	*p*-Cresol	*m*-Cresol	4-Ethylphenol	Syringol	4-Methylsyringol
Panel 1 (Impact of yeast/oak)									
OFV6	5.7 a	0.8 cd	2.5 ab	0.3 ab	1.7 cdef	2.4 ab	2.8 a	54.6 a	8.0 d
EC1118	4.8 cd	0.8 cd	2.5 ab	0.3 b	1.8 bcde	2.4 ab	1.2 a	51.5 ab	8.3 cd
BDX	5.5 a	0.8 cd	2.6 ab	0.4 a	1.5 ef	2.6 ab	1.4 a	53.1 ab	8.3 cd
D80	4.7 d	0.9 bc	2.5 ab	0.3 ab	1.4 f	2.3 b	1.1 a	42.9 e	8.1 cd
D254	5.4 ab	1.0 bc	2.6 a	0.4 ab	2.0 abc	2.6 a	2.8 a	50.7 bc	8.4 cd
NB	5.0 bcd	0.7 d	2.4 b	0.4 ab	2.2 ab	2.4 ab	1.4 a	46.8 d	9.8 b
NF	5.2 abc	1.9 a	2.6 a	0.3 ab	2.4 a	2.6 ab	1.6 a	48.3 cd	8.5 c
QT	4.9 cd	1.0 b	2.6 a	0.3 b	1.9 bcd	2.5 ab	1.3 a	50.6 bc	11.6 a
Unsmoked	2.7 e	0.2 e	1.2 c	0.1 c	1.6 def	0.8 c	1.1 a	37.9 f	6.6 e
Pr > F(Model)	0.000	<0.0001	<0.0001	0.000	0.245	0.036	0.502	<0.0001	<0.0001
Panel 2 (Impact of fermentation temperature/maceration enzymes and tannin)									
AV_25	7.7 a	0.9 b	3.6 a	0.2 c	3.5 bc	4.2 c	2.1 bc	95.3 a	15.6 a
AV_20	7.5 a	0.9 b	3.8 a	0.2 c	4.0 ab	4.8 b	2.2 bc	91.9 b	15.9 a
AV_15	7.7 a	1.0 ab	3.2 ab	0.3 bc	4.4 a	5.5 a	3.3 a	87.1 c	16.0 a
AV_10	6.7 b	0.9 ab	3.5 a	0.4 b	3.5 bc	4.7 b	2.9 ab	65.2 d	14.6 b
ST_C	5.9 c	0.5 cd	1.7 cd	0.3 bc	2.6 d	1.9 e	1.8 c	50.2 e	8.4 c
ST_E+T	7.4 a	0.6 c	2.3 bcd	0.3 bc	3.3 c	1.8 e	1.3 c	49.4 e	8.4 c
S_Control	4.3 d	1.1 a	2.8 abc	0.6 a	2.1 d	2.6 d	2.2 bc	37.9 f	8.1 c
NS_Control	2.6 e	0.3 d	1.611 d	0.247 c	2.1 d	1.0 f	1.4 c	35.4 f	6.4 d
Pr > F(Model)	<0.0001	<0.0001	0.004	0.002	<0.0001	<0.0001	0.004	<0.0001	<0.0001

Significance indicated when letters following values are different within a column for total volatile phenols. No significant difference between fermentation replicates or bottle replicates, thus means displayed.

**Table 3:** Total (free + bound) volatile phenol profiles of samples taken during the winemaking process (n = 3, α ≤ 0.05). All concentrations are in μg/L.

Sample	Guaiacol	Creosol	*o*-Cresol	4-Ethylguaiacol	*p*-Cresol	*m*-Cresol	4-Ethylphenol	Syringol
AV_25								
After one day of cold soak	7.1 d	2.5 b	3.7 bc	14.1 cd	0.3 c	2.9 d	4.9 cd	3.9 b
After 2 days of fermentation	7.9 cd	2.6 b	4.0 b	15.3 c	0.4 b	3.5 d	5.0 cd	4.3 b
After 4 days of fermentation	8.6 bc	2.9 a	4.4 a	17.4 bc	0.6 a	5.2 ab	5.5 bc	4.4 ab
After 7 days of fermentation	9.6 ab	3.1 a	4.7 a	19.9 ab	0.6 a	5.8 a	6.1 ab	4.6 ab
After pressing	10.2 a	3.1 a	4.6 a	22.2 a	0.6 a	5.0 b	6.2 a	5.5 a
AV_20								
After 2 days of fermentation	7.8 b	2.5 b	4.2 b	14.6 b	0.3 b	3.3 c	5.1 b	4.5 a
After 7 days of fermentation	8.7 a	2.7 a	5.1 a	17.4 a	0.4 a	5.6 a	5.4 a	4.6 a
After pressing	8.6 a	2.6 a	5.0 a	17.0 a	0.4 a	4.9 b	5.3 ab	4.6 a
AV_15								
After 2 days of fermentation	7.3 c	2.4 a	3.8 b	13.4 b	0.4 a	2.8 b	4.8 a	4.4 b
After 7 days of fermentation	7.8 b	2.46 a	4.5 a	15.2 ab	0.4 a	3.7 ab	4.8 a	4.3 b
After pressing	8.2 a	2.6 a	4.7 a	16.6 a	0.4 a	4.5 a	5.1 a	4.5 a
AV_10								
After 7 days of cold soak	6.7 a	2.4 a	3.5 b	11.9 b	0.3 b	2.9 a	4.6 a	4.1 a
After pressing	6.9 a	2.2 b	4.0 a	13.8 a	0.6 a	3.5 a	4.4 a	4.1 a
After 7 days of fermentation	6.1 b	1.9 c	3.1 c	8.6 c	0.2 c	2.2 b	3.8 b	3.7 b

Significance indicated when letters following values are different within a column for each volatile phenol measured within a wine treatment. No significant difference between fermentation replicates, thus means displayed.

**Table 4:** Overall means and Fisher’s least significant difference (LSD) for descriptive analysis attributes from panel 1 (n = 3).

Wines	Fig/Dried Fruit	Mineral	Honey	Petrol	Ashy Aftertaste	Sweet	Hot	Astringent	Viscous
BDX_1	2.0	1.1	0.8	1.0	2.3	2.9	3.7	3.7	3.8
BDX_2	3.3	0.8	1.4	0.3	1.3	3.0	2.7	3.2	4.2
D254_1	1.8	0.9	0.8	0.5	2.5	2.4	3.1	4.3	3.5
D254_2	1.9	1.6	1.0	0.7	2.3	2.9	3.2	4.3	3.5
D80_1	2.1	0.8	0.5	0.3	1.6	2.7	3.0	3.6	3.5
D80_2	1.5	1.1	0.8	0.5	2.4	2.3	3.0	3.4	3.1
EC1118_1	2.2	1.2	0.9	0.3	2.6	2.6	3.7	4.0	3.0
EC1118_2	2.5	1.5	0.6	0.5	2.3	2.3	2.7	4.0	3.2
NB_1	2.3	1.0	0.7	0.3	2.4	2.0	3.8	3.9	2.9
NB_2	1.9	1.1	0.8	0.4	2.4	2.2	3.4	3.5	3.5
NF_1	2.0	1.5	0.6	0.3	2.3	1.9	2.4	3.4	2.8
NF_2	2.2	0.9	0.6	0.6	1.7	2.5	3.2	3.9	3.2
OFV6_1	2.1	1.1	0.6	0.4	2.0	2.4	3.5	3.3	3.5
OFV6_2	2.7	0.8	0.7	0.5	2.0	2.3	4.0	3.8	3.6
QT_1	1.9	1.3	0.7	0.3	2.9	1.8	4.2	3.7	3.2
QT_2	2.0	1.5	0.4	0.3	2.0	1.7	3.1	4.0	2.9
Unsmoked_1	3.7	1.0	2.0	0.2	1.1	3.0	2.4	1.2	4.8
Unsmoked_2	2.1	1.0	0.6	0.4	1.5	3.2	2.8	2.0	3.9
LSD-value	0.8	0.5	0.5	0.4	0.9	0.9	1.0	1.1	1.0

**Table 5:** Overall means and Fisher’s least significant difference (LSD) values for significant panel 2 descriptive analysis attributes (n = 3).

Wines	Dark fruit	Vanilla	Green	Smoky	Raisin/Prune	Leather	Hot	Astringent
AV_10.1	3.0	1.3	0.9	1.5	2.3	1.0	4.1	3.0
AV_10.2	2.3	1.7	1.1	1.7	2.1	1.1	3.8	3.2
AV_15.1	2.7	1.6	1.2	1.4	2.0	0.9	4.3	3.4
AV_15.3	1.8	1.3	1.8	1.5	1.4	1.5	4.1	3.7
AV_20.1	3.0	1.6	0.9	1.9	2.0	1.0	3.9	3.6
AV_20.2	2.8	1.8	0.8	1.5	2.4	1.1	4.1	3.6
AV_25.1	2.6	1.8	0.8	1.9	2.1	1.4	4.1	4.4
AV_25.2	2.5	1.6	1.0	1.7	2.4	1.0	4.2	4.4
NS-Control_1	2.7	2.4	0.8	0.9	2.5	0.6	4.6	5.0
NS-Control_2	3.1	1.9	1.2	0.7	2.6	0.6	5.3	5.2
S-Control_1	2.8	2.7	0.6	1.3	2.5	0.7	3.8	3.4
S-Control_2	3.8	2.0	0.4	1.2	3.0	0.7	3.8	4.4
ST_C1	3.7	1.4	0.9	1.4	2.7	1.0	4.3	5.1
ST_C2	3.7	1.6	0.9	1.1	2.7	0.8	4.7	5.1
ST_E+T_1	3.1	1.9	0.6	1.3	2.4	0.9	3.9	6.2
ST_E+T_2	2.2	1.3	1.1	1.0	1.7	1.5	4.4	6.3
LSD-value	0.8	0.7	0.6	0.6	0.7	0.5	0.8	0.7

**Table 6:** Treatment description and wine codes.

Wine code	Vineyard	Treatment
OFV6	Oakville block 6	Standard SOP
EC1118 (Control)	Oakville block 9	Standard SOP, treatment control fermented with yeast EC1118
BDX	Oakville block 9	Fermented with yeast BDX
D80	Oakville block 9	Fermented with yeast D80
D254	Oakville block 9	Fermented with yeast D254
NB	Oakville block 9	Addition of Nobile Base oak chips
NF	Oakville block 9	Addition of Nobile Fresh oak chips
QT	Oakville block 9	Additions of ellagic oak tannin (Quertanin)
Unsmoked	Oakville block 9	Standard SOP, fruit harvested before the wildfires, rootstock 110R
AV_25	Alexander Valley	Standard SOP, treatment control fermented at 25 °C
AV_20	Alexander Valley	Fermented at 20 °C
AV_15	Alexander Valley	Fermented at 15 °C
AV_10	Alexander Valley	Cold soak at 10 °C for 7 days, pressed, fermented without skins/seeds
ST_C	Stag’s Leap District	Standard SOP, treatment control
ST_E+T	Stag’s Leap District	Addition of enzymes and tannin
S-Control	Oakville block 9	Standard SOP from smoke-exposed fruit
NS-Control	Oakville block 9	Standard SOP from non-smoke-exposed fruit harvested before fires rootstock 420A

## Data Availability

Data are contained within the article or supplementary material. The data presented in this study are available in “Investigation of Different Winemaking Protocols to Mitigate Smoke Taint Character in Wine” and supplied Supplementary Material.

## Supplementary Materials

The following supporting information can be downloaded online. Figure S1: MFA map of panel 1 wines’ VP composition and significant sensory attributes, Figure S2: MFA map of panel 2 wines’ VP composition and significant sensory attributes, Table S1: Basic chemical composition of grapes utilized for wines evaluated in panels 1 and 2, Table S2: Air quality index (AQI) and particulate matter (PM) for the periods during which grapes at each site were exposed to prior to harvest, Table S3: Free volatile phenol profiles (n = 6, α ≤ 0.05). All concentrations are in μg/L, Table S4: Bound volatile phenol profiles (n = 6, α ≤ 0.05). All concentrations are in μg/L, Table S5: Panel 1 sensory attributes used for descriptive analysis and corresponding reference standards, Table S6: Correlation matrix for DA of panel 1 (Critical value r Pearson = 0.468; n = 18; 2-tailed; α = 0.05), Table S7: Panel 2 sensory attributes used for descriptive analysis and corresponding reference standards, Table S8: Significant attributes with significant wine x judge interaction and their F-value after applying a pseudo-mixed model for panel 2. Table S9: Correlation matrix for DA of panel 2 (Critical value r Pearson = 0.497; n = 16; 2-tailed; α = 0.05),
