# Supplementary material for: Investigation of Different Winemaking Protocols to Mitigate Smoke Taint Character in Wine
Source: Molecules. 2022 Mar 7;27(5):1732. doi: 10.3390/molecules27051732 (PMC8911878; doi:10.3390/molecules27051732)
Supplement: Supplementary file 1 [file molecules-27-01732-s001.zip › molecules-1537882-supplementary.pdf]

Supplementary materials

Table S1. Basic chemical composition of grapes utilized for wines evaluated in panel one and two.

Grape Code	Vineyard AVA	Harvest date	Brix	pH	*TA (g/L)
		Panel 1			
OFV6	Oakville	October 17th, 2017	24.60 ± 0.26	3.60 ± 0.04	4.29 ± 0.12
OFV9	Oakville	October 17th, 2017	25.00 ± 0.17	3.67 ± 0.01	4.60 ± 0.19
Unsmoked	Oakville	October 7th, 2017	24.27 ± 0.15	3.86 ± 0.04	4.18 ± 0.05
		Panel 2			
AV	Alexander Valley	October 18th, 2017	27.28 ± 0.10	3.82 ± 0.02	3.88 ± 0.04
ST	Stag's Leap District	October 23rd, 2017	24.73 ± 0.21	4.18 ± 0.09	3.60 ± 0.48
S_Control	Oakville	October 17th, 2017	23.97 ± 0.12	3.70 ± 0.01	4.05 ± 0.03
NS_Control	Oakville	October 7th, 2017	$^{\gamma}$22.90 ± 0.01	3.51 ± 0.03	4.80 ± 0.04

*TA = tartaric acid; $^{\gamma}$Grapes were chaptalized to 25˚Brix prior to fermentation.

Table S2. Air quality index (AQI) and particulate matter (PM) for the periods during which grapes at each site were exposed to prior to harvest.

Grape Code	Vineyard AVA	Fire	Smoke exposure period	AQI range	2.5 µm PM (ug/m3)
		Panel 1			
OFV6	Oakville	Tubbs fire (Central LNU Complex)	October 7-17th, 2017	50 - 200	12 – 150.4
OFV9	Oakville	Tubbs fire (Central LNU Complex)	October 7-17th, 2017	50 - 200	12 – 150.4
Unsmoked	Oakville		October 7th, 2017	<50	<12
		Panel 2			
AV	Alexander Valley	Atlas fire (Southern LNU Complex)	October 10-18th, 2017	50 -200	12 – 150.4
ST	Stag's Leap District	Atlas fire (Southern LNU Complex)	October 9-23rd, 2017	50 -300	12 – 250.4
S_Control	Oakville	Tubbs fire (Central LNU Complex)	October 7-17th, 2017	50 - 200	12 – 150.4
NS_Control	Oakville		October 7th, 2017	<50	<12

Table S3. Free volatile phenol profiles (n = 6, $\alpha \leq 0.05$). All concentrations are in µg/L.

Wine	Guaiacol	Creosol	o-Cresol	4-Ethylguaiacol	p-Cresol	m-Cresol	4-Ethylphenol	Syringol	4-Methylsyringol
					Panel 2				
AV_25	5.0 a	0.6 c	3.5 b	0.3 bcd	2.4 b	4.7 c	0.9 b	60.2 ab	1.5 b
AV_20	5.3 a	0.6 b	3.8 a	0.3 bc	2.9 a	5.3 a	1.2 a	56.8 b	1.6 b
AV_15	5.0 a	0.6 b	3.7 a	0.3 b	3.0 a	5.6 a	1.3 a	50.9 c	1.6 b
AV_10	4.5 b	0.6 b	3.2 b	0.3 b	1.5 d	4.0 b	1.0 b	31.4 d	2.0 a
ST_C	4.4 b	0.3 d	2.0 d	0.2 cd	1.7 d	2.4 de	0.6 c	62.2 a	1.1 c
ST_E+T	5.3 a	0.3 de	2.0 d	0.2 d	2.0 c	2.0 e	0.6 c	57.3 b	1.2 c
S_Control	3.1 c	0.9a	2.5 c	0.4 a	0.9 e	2.5 d	0.6 c	50.3 c	2.0 a
NS_Control	2.0 d	0.2 e	1.4 e	0.2 e	0.8 e	0.7 f	0.2 d	49.7 c	0.5 d

Significance indicated when letters following values are different within a column for either free or total volatile phenols. No significant difference between fermentation replicates or bottle replicates, thus means displayed.

Table S4. Bound volatile phenol profiles (n = 6, $\alpha \leq 0.05$). All concentrations are in µg/L.

Wine	Guaiacol	Creosol	o-Cresol	4-Ethylguaiacol	p-Cresol	m-Cresol	4-Ethylphenol	Syringol	4-Methylsyringol
					Panel 2				
AV_25	2.7 a	0.3 ab	0.1 ab	ND c	1.1 b	ND b	1.2 bcd	35.1 a	14.1 a
AV_20	2.3ab	0.3 b	0.1 b	ND c	1.1 b	ND b	1.0 cd	35.1 a	14.3 a
AV_15	2.7 a	0.4 a	0.1 ab	0.0 bc	1.4 ab	0.1 ab	2.0 a	36.2 a	14.4 a
AV_10	2.3 ab	0.3 ab	0.3 ab	0.1 ab	2.0 a	ND b	1.9 ab	33.8 a	12.6 b
ST_C	1.6 cd	0.2 bc	0.1 ab	0.1 abc	0.9 b	ND b	1.2 bcd	ND b	7.3 c
ST_E+T	2.1 bc	0.3 ab	0.3 ab	0.1 bc	1.3 b	ND b	0.7 d	ND b	7.2c
S_Control	1.2 d	0.2 bc	0.4 a	0.2 a	1.2 b	0.1 ab	1.6 abc	ND b	6.0 d
NS_Control	0.6 e	0.1 c	0.2 ab	0.1 abc	1.4 ab	0.3 a	1.3 abcd	ND b	5.9 d

Significance indicated when letters following values are different within a column for either free or total volatile phenols. No significant difference between fermentation replicates or bottle replicates, thus means displayed. ND = not detected.

Table S5. Panel one sensory attributes used for descriptive analysis and corresponding reference standards.

**Aroma**	**Reference Standards**
**alcohol**	20 mL ethanol (151 proof Everclear, Luxco) + 30 mL base wine
**strawberry**	1 thawed strawberry cut into pieces (Full circle market organic whole strawberries)
**dark fruit**	3 crushed Signature Kitchen dark sweet, pitted cherries + 1 Signature Select blackberry + 4 Signature Select blueberries + 10 mL Ribena blackcurrant juice + 10 mL base wine
**red cherry**	3 chopped Signature Kitchen dark sweet, pitted cherries + 5 mL Signature Select Maraschino cherries juice + 1 teaspoon Signature Kitchens cherry preserves
**citrus**	2 drops citrus essence (Bell Flavors & Fragrances, Inc. natural citrus flavor #123.9065) + 3 g of chopped orange (flesh, no zest) +20 mL base wine
**fig/dried fruit**	2 crushed Trader Joe's black mission dried figs + 10 mL base wine
**mineral**	5 rocks + 20 mL of tap water
**spices**	1 pinch McCormick ground nutmeg + mini pinch McCormick ground clove + 50 mL base wine
**black pepper**	1/8 tsp McCormick pure ground black pepper + 10 mL base wine
**floral**	3 drops Carlo rose water + 4 drops Sadaf orange blossom water + 1 drops Bigallet violet syrup+ 50mL of base wine
**wood**	5g Nobile American oak chips + 0.1g cedar wood chips (PTCLTRAPS Drawer Essentials America red cedar sachet natural clothing deodorizer) + 0.2 g wood shavings + 0.1 g pencil shavings + 20 mL base wine
**moldy/dusty**	10 g Crayola Air-Dry clay + 20 mL tap water + 2 g of ripped cardboard
**vanilla**	4 drops Madecasse pure vanilla extract + 20mL base wine
**vinegar**	1/2 tsp Signature Kitchens distilled white vinegar + 20mL base wine
**leather**	leather shoelace (all of it)
**green**	fresh cut grass from lawn + 5 g chopped bell pepper + pinch McCormick celery salt + 10 mL tap water
**soil/earthy**	1/2 Tbsp Miracle Gro potting soil + 10mL tap water
**herbs (Provence)**	1/8 tsp McCormick Gourmet herbs de Provence + 20mL base wine
**honey**	1 Tbsp of Signature Kitchens clover honey
**petrol**	0.4 mL Kingsford odorless charcoal lighter + 50mL base wine
**menthol**	10 mL Vick's Nyquil cold & flu syrup + 3 drops eucalyptus water (1 drop euc oil in 50 mL water, Nature's Alchemy Eucalyptus Essential Oil) + 30 mL base wine
**smoke**	4 drops Colgin liquid smoke + 20mL base wine
**anise**	1/8 tsp of McCormick anis seeds
**ashy (aftertaste)**	2 g of burnt Lazzari mesquite smoking chips + 5mL water + 0.5g cigarette ash (1 Marlboro)
**hot**	350 mL/L ethanol (151 proof Everclear, Luxco) in water
**viscous**	3 g/L of Carboxymethyl Cellulose (CMC, Sigma-Aldrich) in water
**astringent**	5 g/L of alum (McCormick) in water

**sour**	3 g/L L-(+)-tartaric acid (Fisher Scientific) in water
**bitter**	3 g/L caffein (Sigma-Aldrich) in water
**sweet**	15 g/L sucrose (C&H pure cane sugar) in water

Notes: Franzia chillable red wine was used as base wine.

Table S6. Correlation matrix for DA of panel one (Critical value r Pearson = 0.468; n=18; 2-tailed; α=0.05).

Variables	Fig/dried fruit	Mineral	Honey	Petrol	Ashy aftertaste	Sweet	Hot	Astringent	Viscous
Fig/dried fruit	**1**								
Mineral	-0.368	**1**							
Honey	0.723	-0.235	**1**						
Petrol	-0.334	0.117	-0.148	**1**					
Ashy aftertaste	-0.673	0.485	-0.443	0.185	**1**				
Sweet	0.366	-0.413	0.496	0.278	-0.600	**1**			
Hot	-0.311	-0.114	-0.293	0.218	0.579	-0.328	**1**		
Astringent	-0.555	0.281	-0.569	0.336	0.710	-0.476	0.476	**1**	
Viscous	0.656	-0.430	0.758	0.061	-0.677	0.755	-0.281	-0.707	**1**

Notes: Green indicates a significant positive correlation whereas red indicates a significant negative correlation.

Table S7. Panel 2 sensory attributes used for descriptive analysis and corresponding reference standards.

Aroma	Reference Standards
**alcohol**	30 mL Borski vodka + 30 mL base wine
**strawberry**	1 thawed strawberry cut into pieces (Full circle market organic whole strawberries)
**dark fruit**	1 crushed Signature Kitchen dark sweet, pitted cherry + 1 Oregon Specialty fruit red tart cherry + 1 Signature Select blackberry + 5 Signature Select blueberries + 1g fresh plum + 15mL Ribena blackcurrant juice + 15mL base wine
**vanilla**	1 Tbsp of Snack Pack sugar free vanilla pudding + 20 mL base wine
**raspberry**	3 crushed Signature Select raspberries + 10 mL of base wine
**citrus**	1/2 tsp St Dalfour fruit spread orange marmalade + 1sq" fresh orange peel + 0.5sq" fresh lime peel + 50mL base wine
**red apple**	10 g fresh Golden delicious apple chopped + 10 mL Martinelli's apple cider
**green**	3 g chopped fresh green bell pepper + 20 mL base wine
**mineral**	3 big rocks and 10 small rocks + 5mL of tap water
**spices**	1 small pinch blend (equal parts ground: McCormick ground cinnamon + McCormick ground all spice + McCormick ground clove + McCormick ground nutmeg + McCormick ground ginger) + 30mL base wine
**pepper**	1/8 tsp McCormick ground black pepper + 1 small pinch McCormick ground white pepper + 30mL base wine
**floral**	3 drops Carlo rose water + 2 drops Sadaf orange blossom water + 1 drop Bigallet violet syrup + 10mL The Republic of Tea hibiscus tea + 30 mL of base wine
**oak/wood**	2 g Nobile Fresh oak chips + 0.2 g cedar wood chips (PTCLTRAPS Drawer Essentials America red cedar sachet natural clothing deodorizer) + 0.5 g wood shavings + 20mL base wine
**ash (aftertaste)**	2 g of burnt Lazzari mesquite smoking chips + 5mL water + 0.5g cigarette ash (1 Marlboro)
**pipe tobacco**	1 g Stave-aged Virginia 35 ribbon pipe tobacco
**smoky**	4 drop Colgin liquid smoke + 20 mL base wine
**leather**	leather shoelace (all of it)
**pineapple**	5 g fresh pineapple chopped
**raisins/prune**	15 mL Sunsweet prune juice + 10 Sun-maid California sun-dried smashed raisins + 10 mL base wine
**earthy**	1/2 Tbsp fresh soil + 10mL tap water
**vinegar**	1/4 tsp Signature Kitchens distilled white vinegar + 25 mL base wine
**hot**	325 mL/L ethanol (151 proof Everclear, Luxco) in water
**viscous**	3 g/L of Carboxymethyl Cellulose (CMC, Sigma-Aldrich) in water
**astringent**	5 g/L of alum (McCormick) in water
**sour**	1 g/L L-(+)-tartaric acid (Fisher Scientific) in water
**bitter**	1 g/L caffein (Sigma-Aldrich) in water
**sweet**	15 g/L sucrose (C&H pure cane sugar) in water

Notes*: Franzia chillable red wine was used as base wine.*

Table S8. Significant attributes with significant wine × judge interaction and their F-value after applying a pseudo-mixed model for panel two.

SIGNIFICANT ATTRIBUTE	F-VALUE AFTER PSEUDO-MIXED MODEL[1]
**STRAWBERRY**	1.55851919
**DARK FRUIT**	2.68232707
**SPICES**	1.42301731
**LEATHER**	1.65828321
**EARTHY**	1.55634269
**ASH (AFTERTASTE)**	1.30510675
**SOUR**	1.25659272
**SWEET**	1.29216204
**ASTRINGENT**	11.5400184
**VISCOUS**	1.54855244

[1] =F for 15 and 165 at 5% is between 1.85 and 1.59

Table S9. Correlation matrix for DA of panel two (Critical value r Pearson = 0.497; n=16; 2-tailed; α=0.05).

Variables	Dark fruit	Vanilla	Green	Smoky	Raisins/prune	Hot	Astringent	Leather
Dark fruit	**1**							
Vanilla	0.197	**1**						
Green	-0.614	-0.589	**1**					
Smoky	-0.284	-0.320	0.029	**1**				
Raisins/prune	0.872	0.499	-0.694	-0.377	**1**			
Hot	0.137	-0.079	0.321	-0.669	0.162	**1**		
Astringent	0.230	-0.003	-0.168	-0.574	0.178	0.454	**1**	
Leather	-0.630	-0.636	0.436	0.493	-0.777	-0.284	0.003	**1**

Notes: Green indicates a significant positive correlation whereas red indicates a significant negative correlation.

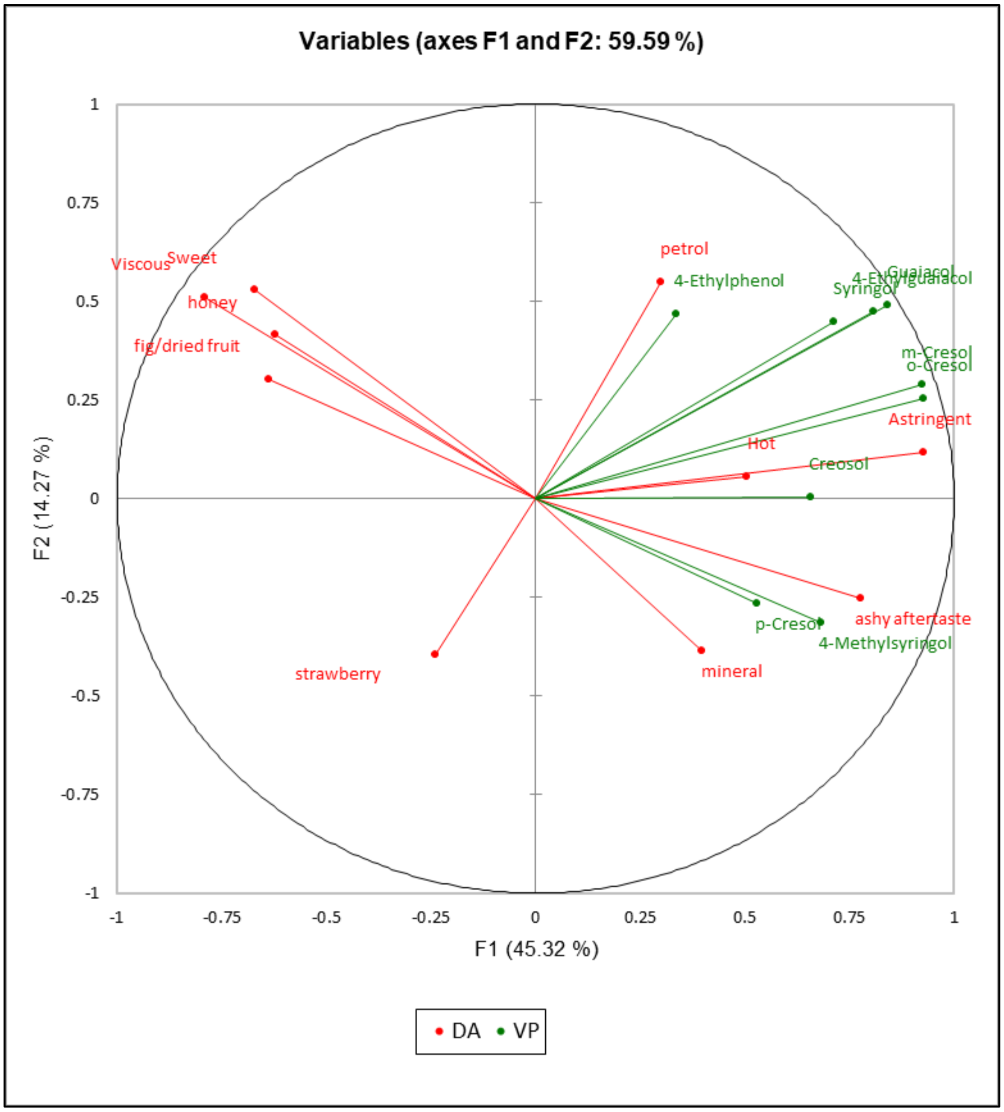

Figure S1. MFA map of panel one wines' VP composition and significant sensory attributes.

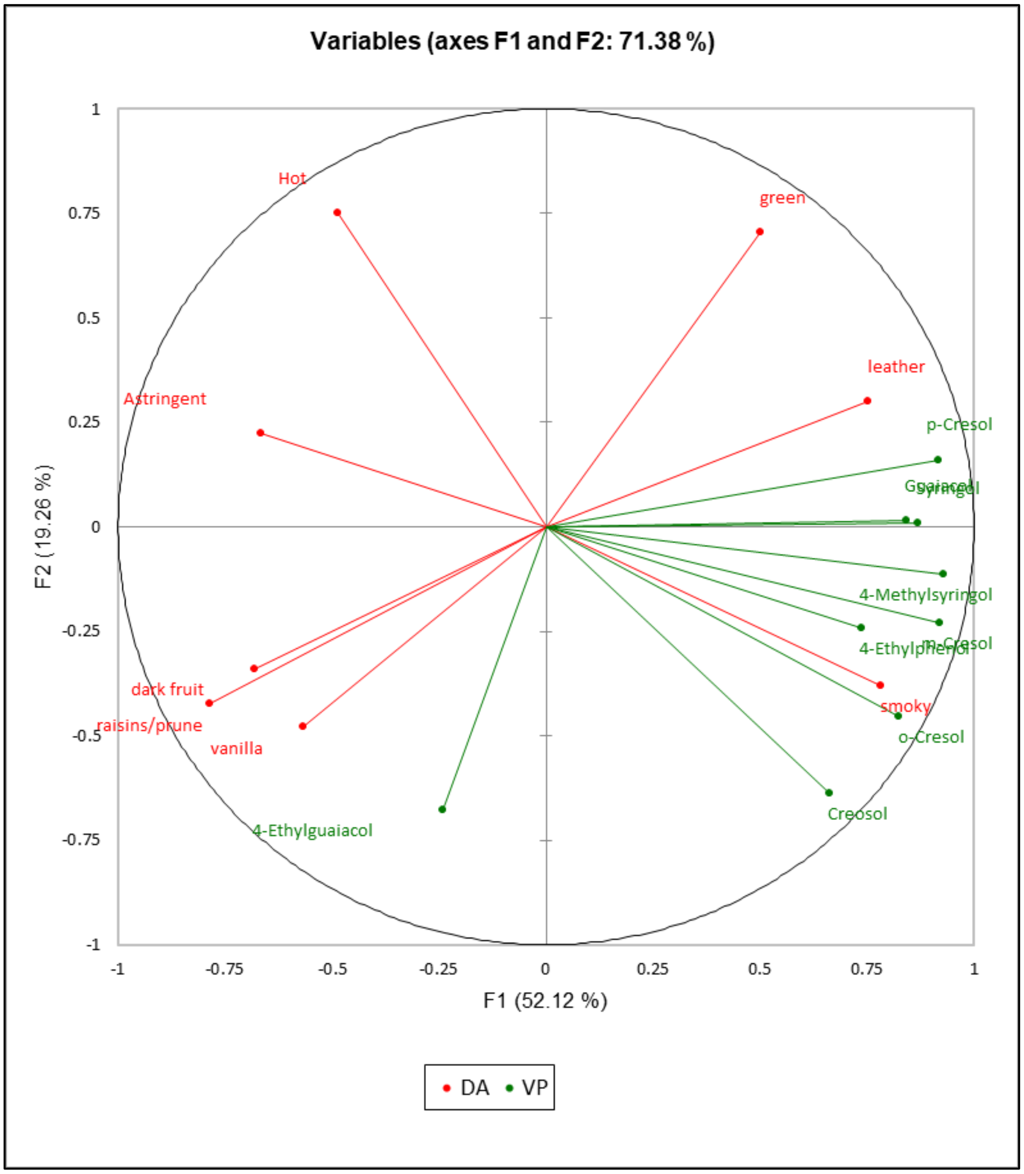

Figure S2. MFA map of panel two wines' VP composition and significant sensory attributes.
